# Epigallocatechin-3-gallate (EGCG) attenuates non-alcoholic fatty liver disease via modulating the interaction between gut microbiota and bile acids

**DOI:** 10.3164/jcbn.20-39

**Published:** 2020-06-03

**Authors:** Yuji Naito, Chihiro Ushiroda, Katsura Mizushima, Ryo Inoue, Zenta Yasukawa, Aya Abe, Tomohisa Takagi

**Affiliations:** 1 Molecular Gastroenterology and Hepatology, Kyoto Prefectural University of Medicine, 465 Kajii-cho, Kamigyo-ku, Kyoto 602-8566, Japan; 2 Department of Endoscopy and Ultrasound Medicine, University Hospital, Kyoto Prefectural University of Medicine, 465 Kajii-cho, Kamigyo-ku, Kyoto 602-8566, Japan; 3 Laboratory of Animal Science, Setsunan University, Nagaotoge-cho 45-1, Hirakata, Osaka 573-0101, Japan; 4 Nutrition Division, Taiyo Kagaku Co., Ltd., 1-3 Takaramachi, Yokkaichi, Mie 510-0844, Japan; 5 Department for Medical Innovation and Translational Medical Science, Kyoto Prefectural University of Medicine, 465 Kajii-cho, Kamigyo-ku, Kyoto 602-8566, Japan

**Keywords:** dysbiosis, epigallocatechin-3-gallate, high-fat diet, taurine, nutrigenomics

## Abstract

The spectrum of non-alcoholic fatty liver disease (NAFLD) ranges from simple hepatic steatosis commonly associated with obesity, to non-alcoholic steatohepatitis, which can progress to fibrosis, cirrhosis and hepatocellular carcinoma. Recent reports have indicated the crucial role of gut microbiota and their metabolites in the progression of NAFLD. In the present review, we demonstrated the influence of oral administration of (−)-epigallocatechin-3-gallate (EGCG) on the gut microbiota, serum bile acid profile, and gene expression in the liver in mice fed a high-fat diet (HFD). EGCG significantly inhibited the increase in histological fatty deposit and triglyceride accumulation in the liver induced by HFD, and improved intestinal dysbiosis. One of important findings is that the abundance of Proteobacteria and Defferibacteres phylums increased markedly in the HFD group, and this increase was significantly suppressed in the EGCG group. Interestingly, taurine-conjugated cholic acid (TCA) increased in the HFD group, like the mirror image against a marked decrease in the cholic acid (CA) value, and this increase was markedly inhibited in the EGCG group. TCA is not a simple serum biomarker for liver injury but TCA may be a causal factor to disturb lipid metabolism. The distribution of correlation coefficients by Heatmap analysis showed that the abundance of *Akkermansia* and *Parabacteroides* genus showed a positive correlation with CA and a negative correlation with TCA, and significantly increased in the EGCG group as compared with the HFD group. In addition, nutrigenomics approaches demonstrated that sirtuin signaling, EIF2 pathway and circadian clock are involved in the anti-steatotic effects of EGCG. In the present paper, we summarized recent update data of EGCG function focusing on intestinal microbiota and their interaction with host cells.

## Introduction

Non-alcoholic fatty liver disease (NAFLD) is an important public health issue because of its high prevalence. The spectrum of NAFLD ranges from simple hepatic steatosis to non-alcoholic steatohepatitis (NASH), which can progress to fibrosis, cirrhosis, and hepatocellular carcinoma (HCC).^(1)^ Recent studies are gradually revealing the abnormalities of the gut microbiota and their metabolites in patients with NAFLD^(2,3)^ as well as in an animal model induced by high-fat diet (HFD).^(4,5)^ Recent metagenomic analysis by the next generation sequencers has demonstrated that Proteobacteria phylum is closely involved in hepatic fibrosis of NAFLD patients,^(6)^ and that *Bilophila wadsworthia*, a genus of Proteobacteria phylum, could aggravate HFD-induced inflammatory response.^(7)^

In addition, when considering the mechanism by which dysbiosis of the resident microbiota could modify the pathology of NAFLD, several studies have revealed an important role of metabolites produced by microbiota. Gut microbes could produce many products such as short-chain fatty acids, trimethylamine, ammonia, and hydrogen peroxides. Interestingly, Caussy *et al.*^(8)^ has reported an evidence of a link between hepatic steatosis/fibrosis and the gut microbiome-derived metabolite 3-(4-hydroxyphenyl) lactate, which is a product of aromatic amino acids metabolism by a prospective cohort study. In recent years, several metabolites of bile acids (BAs) have been shown to regulate lipid and carbohydrate metabolism as well as energy homeostasis in both hepatic and extrahepatic tissues, through regulating the activation of BA-specific receptors, farnesoid X receptor (FXR) and transmembrane G protein-coupled receptor (TGR)-5, positively and negatively.^(9)^

Recently, Ushiroda *et al.*^(10)^ have demonstrated that serum taurine-conjugated BAs increased in HFD-fed mice by the quantitative systematic liquid chromatography-tandem mass spectrometry (LC-MS/MS) and that the correlation analysis between BAs profiles and gut microbiota demonstrated their contribution on the modulation of BAs metabolism induced by the treatment with (–)-epigallocatechin-3-gallate (EGCG), the most abundant polyphenolic catechin in green tea. The objectives of this paper are to review and discuss the currently known targets, microbiota and BAs, and role of EGCG that interfere with NAFLD pathogenesis.

## Prevention of NAFLD by EGCG

Taguchi *et al.*^(11)^ have investigated the associations between subjects’ total polyphenol intake and their mortality from all causes, cardiovascular disease, cancer, and other causes of death in a population-based cohort study in a total of 29,079 residents of Takayama City. They showed that the highest quartile of total polyphenol intake compared with the lowest quartile was significantly associated with a lower risk of all-cause mortality after multivariable adjustment. Some research groups have conducted a few clinical trials to examine the efficacy and safety of EGCG or green tea extract (GTE) rich in EGCG on the subjects with NAFLD, NASH, or obesity. Fukuzawa *et al.*^(12)^ have demonstrated that patients with NASH who were diagnosed by liver biopsy took 600 mg of GTE per day for 6 months with controlled diets and exercise therapy achieved significant effects, including significant decrease in body mass index and improved lipid/glucose profiles. Meanwhile, the high level of high sensitive-CRP was decreased, the ALT and AST levels remained under the limits for most of the patients in the GTE treatment group. Although increasing evidences that EGCG possesses broad biological effects that may be useful in the prevention or treatment of NAFLD, no randomized, controlled, clinical trials have tested the effects of EGCG or GTE on human NAFLD/NASH at this time.^(13)^

**Fig. 1:**
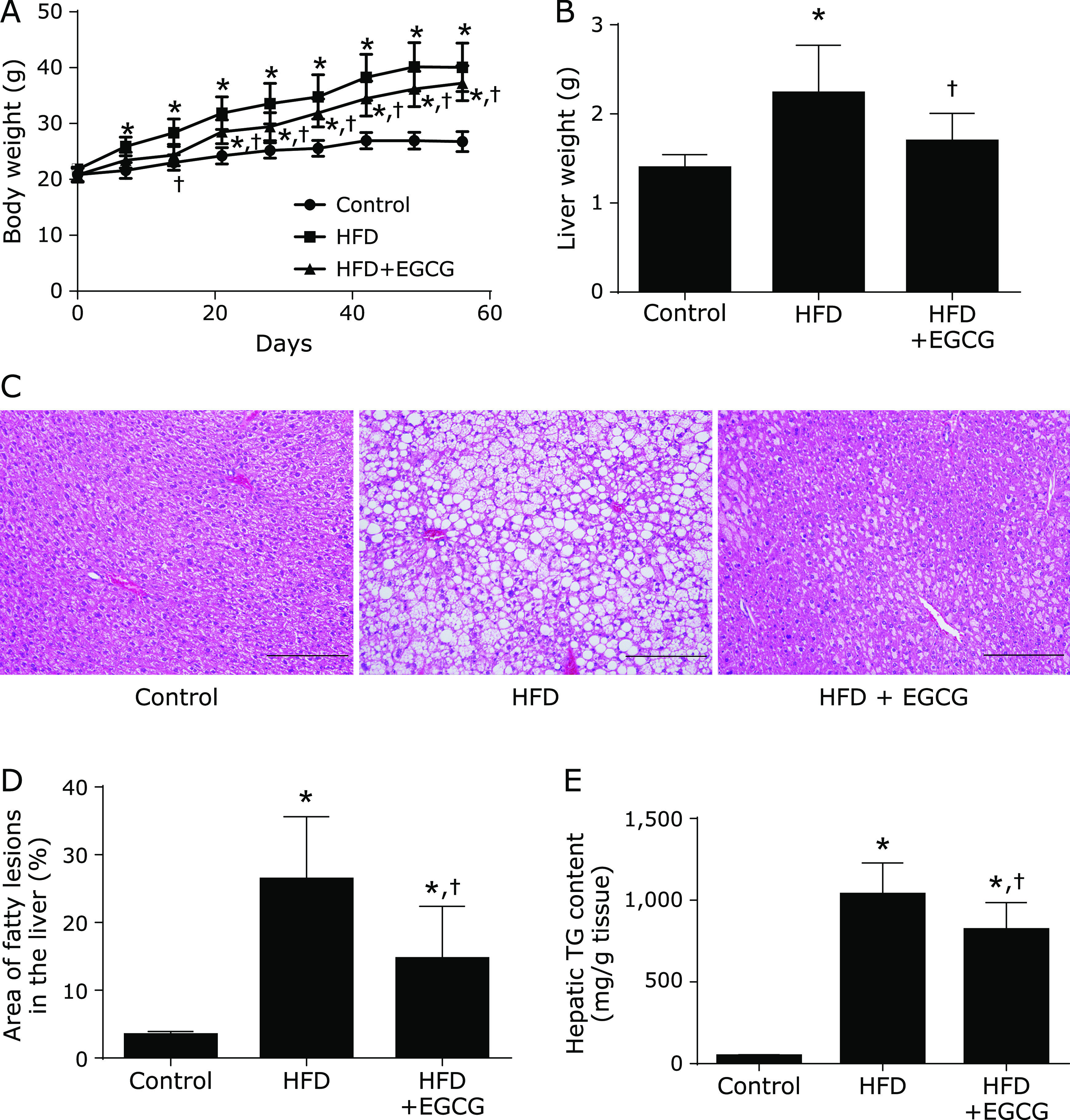
Inhibitory effect of EGCG on the HFD-induced obese phenotype and hepatic triacylglycerol accumulation. (A) Time course of mouse body weight. Body weight of male mice receiving an EGCG containing diet for 8 weeks. (B) Liver weight. (C) Hematoxylin eosin (HE)-stained liver sections. (D) Area of fatty lesions in the liver. (E) Hepatic triacylglycerol (TG) content. C57BL/6N mice were fed a CE2 diet (control), a high-fat diet (HFD), or an HFD supplemented with 0.32% EGCG (HFD + EGCG) for 8 weeks. Values are expressed as the means and SEM of eight mice in each group. Significant differences compared with the control group are denoted *p<0.05, and those with the HFD group are denoted ^†^*p*<0.05. Photographs are of HE staining of liver sections from representative mice of each group (scale bar = 500 mm). See color figure in the on-line version. Published with permission.^(10)^

EGCG has been widely investigated in terms of its health benefits, including anti-steatotic properties on the liver. Previous studies have demonstrated that the treatment with EGCG suppressed fat deposition in the liver in high-fat diet (HFD)-fed mice, a murine model of human NAFLD, via the regulation of intracellular second messengers, signal transduction pathways, transcriptional activation, and autophagy pathway.^(13)^ We recently reported that EGCG significantly inhibited the increase in histological fatty deposit and triglyceride (TG) accumulation in the liver induced by HFD (Fig. 1).^(10)^ Three groups of eight mice each fed one of the following diets: control (CE-2; CLEA Japan, Tokyo, Japan), an HFD (HFD32; CLEA Japan), or an HFD with EGCG for 8 weeks. EGCG (Sunphenon^®^, Taiyo Kagaku Co., Ltd., Mie, Japan) was supplemented in the food at a concentration of 0.32%. In the study, the dose of EGCG used (0.32%) corresponded to the consumption of approximately 10 cups of green tea per day in humans. The final liver weight at the end of the experiment was significantly increased in the HFD group compared to the control group, and this increase was significantly inhibited by the treatment with EGCG (Fig. 1B). Although the histological liver sections of the control group were free of lipid droplets, increased accumulation of lipid droplets was observed in the HFD group, leading to a condition of hepatic steatosis, and this increase was suppressed in the HFD + EGCG group. Consistent with the histological findings, the area of fatty lesions in the liver was significantly increased in the HFD group compared to the control, and this increase was significantly inhibited by the treatment with EGCG (Fig. 1D). In addition, the content of TG in the liver was significantly increased in the HFD group, and this increase was also inhibited by the treatment with EGCG (Fig. 1E). In addition to anti-steatotic effects in animal models of NAFLD, recent reports also have demonstrated that a series of fibrosis signaling pathways in the liver were down-regulated by EGCG treatment,^(14)^ and that EGCG prevents obesity-related liver tumorigenesis by inhibiting the IGF/IGF-1R axis and attenuating chronic inflammation.^(15)^ Although the efficacy of EGCG for NAFLD with a wide range of stages from fatty liver to liver cancer is clear in animal models, it is regretted that no clinical studies have been completed on humans.

## Dysbiosis in NAFLD

The possibility that the functionality of EGCG could be derived from the influence on the gut microbiota and intestinal environment has recently attracted attention, since catechins including EGCG have extremely low absorbability from the intestinal tract. Actually, it has been reported that EGCG reduced the occupation of *Clostridium spp.* and tends to increase *Bacteroides* in rats.^(16)^ Recently, Sheng *et al.*^(17)^ has reported that EGCG increased the abundance of *Akkermansia muciniphila* bacteria and promoted the release of glucagon-like peptide (GLP)-1 from intestinal tract via the activation of TGR-5 in mice fed a Western diet. We also showed that principal coordinate analysis (PCoA) showing β-diversity clearly distinguished control, HFD and HFD + EGCG mice groups.^(10)^ At the phylum level, the relative abundance of Deferribacteres and Proteobacteria were significantly increased in the HFD group as compared to control, and EGCG significantly reversed these changes. The Firmicutes/Bacteroidetes ratio, an index of dysbiosis, tended to increase in the HFD group as compared to control, and this increase was significantly inhibited in the EGCG group. These results suggested that EGCG improved dysbiosis of gut microbiota induced by HFD and, as a result, it may be involved in the anti-fatty liver action of EGCG.

One important finding in our study is that the abundance of Proteobacteria phylum increased markedly in the HFD group compared with the control group, and this increase was significantly suppressed in the EGCG group. *Bilophila wadsworthia* belonging to p_Proteobacteria; f_Desulfovibrionaceae; g_*Bilophila* was identified as a bacterium exacerbating metabolic disorder caused by HFD, and it has been demonstrated that *Bilophila wadsworthia* bacteria aggravates the metabolic dysfunction in HFD-fed mice by enhancing intestinal mucosal permeability, promoting inflammatory immune response, and altering BA metabolism.^(7)^ Although g_*Bilophila* was hardly detected in our study, p_Proteobacteria; f_Desulfovibrionaceae; g_*Unclassified*, which is a related genera, increased more than 10-fold in the HFD group as compared with the control group, and EGCG significantly inhibited this increase. These results suggest that f_Desulfovibrionaceae may play a crucial role in the development of HFD-induced fatty liver in this rodent model.

In our study, the most striking feature was the significant increase of the *Akkermansia *genus. Recently, Sheng *et al.*^(17)^ reported that *Akkermancia* genus is significantly increased by Western diet with EGCG administration. *Akkermansia muciniphila* is the main genus classified in the Verrucomicrobia phylum and in recent studies this bacteria has been revealed to be involved in obesity, sugar metabolism, and intestinal immunity.^(18)^ In previous studies, cranberry extracts,^(19)^ concord grape polyphenols,^(20)^ and apple procyanidin^(21)^ have been interestingly reported to show an anti-obesity effect through an increased abundance of the *Akkermansia* genus. In addition, these effects have also been reproduced by the administration of *Akkermansia muciniphila* to mice.^(22)^ Such polyphenols, including EGCG, generally have low absorption from the intestinal tract and reach the large intestine without being digested and absorbed, but details of the mechanism of increasing *Akkermansia* have not been elucidated. It is thought that *Akkermansia muciniphila* produces short-chain fatty acids such as acetic acid by feeding intestinal mucin, and supplies energy to goblet cells that produce mucin.^(23)^ In our experiment, it is suggested that *Akkermansia* genus markedly increased by EGCG may be involved in hepatoprotection via various mechanisms. *Akkermansia* genus in feces may be one of the good surrogate markers to consider the functionality of EGCG. A further human clinical trial is necessary to confirm the functionality of EGCG focusing on gut microbiome.

## Interaction between Bile Acids and Gut Microbes in NAFLD

**Fig. 2:**
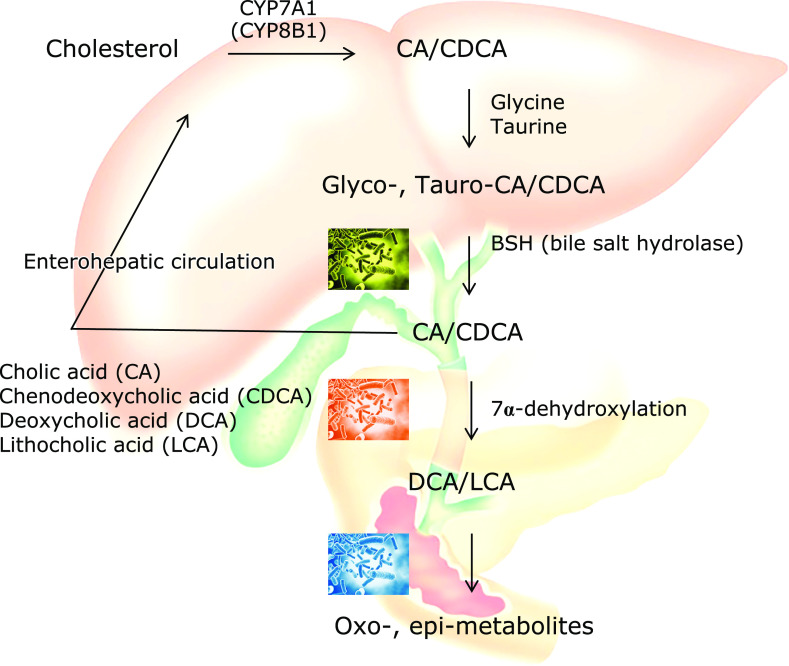
Bile acids synthesis, conjugation, and metabolism associated with gut microbiota.

Primary BAs synthesized in human liver are cholic acid (CA) and chenodeoxycholic acid (CDCA), conjugated with glycine or taurine (Glyco-CA, Glyco-CDCA, Tauro-CA, Tauro-CDCA), and they are excreted into the bile. In the terminal ileum and the colon, bile salt hydrolase (BSH) expressed in various bacteria that deconjugate glycine and taurine (Fig. 2). The hydroxyl group at the C-7α position of the deconjugated BAs is then dehydroxylated to form secondary BAs, deoxy-cholic acid (DCA) and lithocholic acid (LCA), by multi-step reactions of specific bacteria.^(24)^ In addition, hydroxyl groups at the C-3α, 7α, and 12α positions of both conjugated and unconjugated BAs can be dehydrogenated to carbonyl groups and further epimerized to 3β-, 7β-, and 12β-hydroxyl groups by intestinal bacteria. In mice, α-muricholic acid (α-MCA) and β-MCA are produced in the liver from CDCA as a primary BA, and these BAs were also conjugated by glycine and taurine and dehydroxylated to form ω-MCA as a secondary BA.

However, it is also important to understand that bile acid metabolism differs between humans and mice. First, most of CDCA, an end product in human liver, is further metabolized to muricholic acid (MCAs) by CDCA 6β-hydroxylase in liver of mouse and rat. CDCA is known to be the most potent physiological agonist of the FXR, in contrast, MCAs have antagonistic effects of FXR. Second, CA and CDCA are 7α-dehydroxylated by the intestinal bacteria and transformed into the secondary BAs. In mice and rats, these secondary BAs are converted to primary BAs by the hepatic BA 7α-hydroxylase. Honda *et al.*^(25)^ recently generated double knock-out mice of genes encoding CDCA 6β-hydroxylase and BA 7α-hydroxylase to examine BA metabolism. These double knock-out mice could be a useful model for investigating the role of BAs in various human disease.

**Fig. 3:**
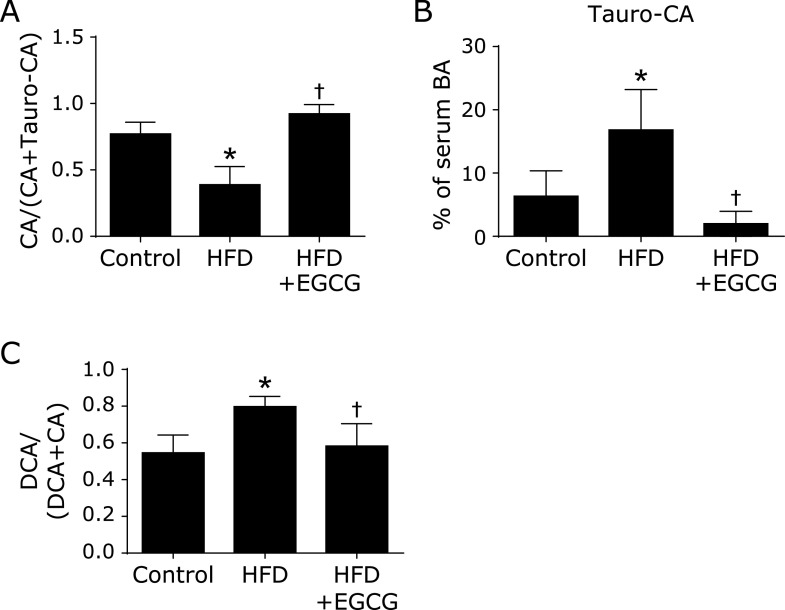
Determination of BA profile and calculation of BA transformation activities by gut microbiota, and the effects of EGCG on these changes. (A) Deconjugation ratio of tauro-cholic acid (Tauro-CA), (B) Tauro-CA in serum, and (C) 7α-dehydroxylation ratio of deoxycholic acid (DCA). C57BL/6N mice were fed with the control CE-2 diet (control), a high-fat diet (HFD), or the HFD supplemented with 0.32% EGCG (HFD + EGCG) for eight weeks. Values are expressed as the means + SE of six mice in each group. *p<0.05, significant differences compared with the control group, and ^†^*p*<0.05 those with the HFD group. Published with permission.^(10)^

Our data from the serum BA profiles determined by LC-MS/MS showed that HFD significantly decreased the level of total free BAs, and the reduction was significantly reversed by EGCG supplementation. Among free BAs, CA, ω-MCA, ursodeoxycholic acid (UDCA), ursocholic acid (UCA), hyodeoxycholic acid (HDCA), and 7-oxodeoxycholic acid (7-oxo DCA) were significantly decreased in the HFD group compared to the control group. The HFD-induced decrease in CA, a predominant primary BA in mice, was significantly reversed by the treatment with EGCG. Contrary to the changes in free BAs, HFD increased the total level of taurine-conjugated BAs, and this increase was significantly inhibited by the treatment with EGCG. Among taurine-conjugated BAs, Tauro-CA and Tauro-DCA were significantly increased in the HFD group compared to the control, and these increases were significantly inhibited by EGCG (Fig. 3B).

Deconjugation by gut microbiota was calculated, CA/(CA + Tauro-CA) ratio, was significantly decreased in the HFD group. The HFD-induced decreases in the deconjugation ratio was significantly reversed by the treatment with EGCG (Fig. 3A). 7α-Dehydroxylation by gut microbiota was calculated by DCA/(DCA + CA) ratio and this ratio was significantly increased in the HFD group compared to the control, this increase was significantly inhibited by the treatment with EGCG (Fig. 3C). These data suggest that EGCG increases serum levels of CA and CDCA through improving the intestinal environment, leading the improvement of liver lipid metabolism and the inhibition of fatty liver. Furthermore, Tauro-CA increased markedly in the HFD group as compared with the control group, like the mirror image against a marked decrease in the CA value. The reduction of serum taurine-conjugated BAs by EGCG appears to be additional important mechanisms explaining the anti-fatty liver action of EGCG. The significant role of serum conjugated BAs in the progression of NAFLD has been recently demonstrated by a cross-sectional analysis in patients with biopsy-proven NAFLD.^(26)^ They demonstrated that the total serum BAs did not differ significantly among non-NAFLD, NAFLD, and NASH, and that the heat map of the spearman correlation showed the positive correlation between conjugated CA and the progression of NAFLD, and the negative correlation between unconjugated CA and NAFLD progression. Although there is not enough evidence on how taurine-conjugated BAs contribute to the pathology of NAFLD, clinical evidence is recently accumulating by BA profiling analyses. Future research is needed.

**Fig. 4:**
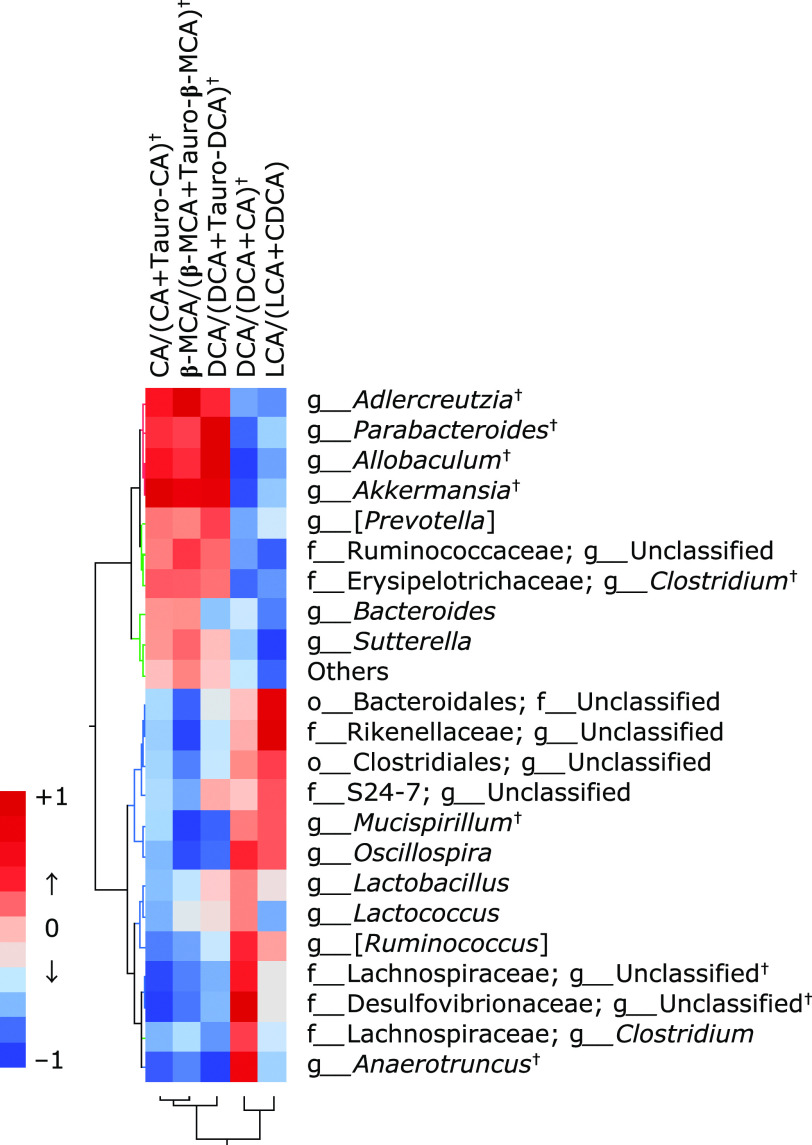
Correlation heat map demonstrating the association between the indicated gut microbiota taxonomic genera and BA transformation index, including deconjugation ratio and 7α-dehydroxylation by gut microbiota. Significant positive correlations were observed between serum CA/(CA + Tauro-CA) and *Akkermansia*, *Allobaculum*, *Adlercreutzia*, or *Parabacteroides*, and between DCA/(DCA + CA) and f_*Desulfovibrionaceae*; g_*Unclassified*, *Anaerotruncus*, f_*Lachnospiraceae*; g_unclassified, or [*Ruminococcus*]. ^†^*p*<0.05 compared between the high-fat diet group and the high-fat diet + EGCG group. Published with permission.^(10)^

From the correlation between microbiota abundance obtained by 16S rRNA metagenomic analysis and serum BA profiles obtained by quantitative LC-MS/MS, we further examined the functionality of EGCG from the standpoint of the interaction of these markers. Judging from the distribution of correlation coefficients by Heatmap analysis, the abundance of *Akkermansia* and *Parabacteroides* genus showed a positive correlation with CA and a negative correlation with Tauro-CA, and significantly increased in the EGCG group as compared with the HFD group. As we suspected that Tauro-CA was recovered in the EGCG group due to the deconjugation reaction of Tauro-CA by intestinal bacteria with the bile salt hydrolase (*bsh*) gene, we searched for genera positively correlated with the ratio of CA/CA + Tauro-CA, β-MCA/β-MCA + Tauro-β-MCA, and DCA/DCA + Tauro-DCA, as indices of deconjugation of BAs. As a result, the abundance of *Akkermansia* and *Parabacteroides* genera showed significant and strong correlation with the deconjugation indices (Fig. 4). These results indicate that the increased abundance of *Akkermansia* and/or* Parabacteroides* may be involved in promoting the taurine deconjugation reaction from taurine-conjugated BAs to free type of BAs.

Since the production of secondary BA depends on the 7α-hydroxylation gene of gut microbiota, heatmap analysis was also performed on the abundance of microbiota correlated with the ratio of DCA/DCA + CA and LCA/LCA + CDCA, as indices of 7α-hydroxylation of BAs. We also investigated the correlation between BA transformation index and the abundance of gut microbiota (Fig. 4). Five genera were chosen as bacteria significantly correlated with the 7α-hydroxylation ratio; f_*Lachnospiraceae*; g_*unclassified*, g_[*Ruminococcus*], and g_*Oscillospira* with moderate positive correlation and g_*Anaerotruncus* and f_*Desulfovibrionaceae*; g_unclassified with strong positive correlation. Among the five genera, f_*Desulfovibrionaceae*; g_Unclassified is high abundance in the HFD group, suggesting the possibility that this bacterium was key in the production of DCA, but it is unknown how much it contributes to secondary BA production.

These data suggested that serum taurine-conjugated BAs (Tauro-CA, Tauro-β-MCA, and Tauro-DCA) increased in HFD-fed mice and that the correlation analysis between BA profiles and gut microbiota demonstrated the contribution of *Akkermansia* and f_*Desulfovibrionaceae*; g_unclassified in the modulation of BA metabolism induced by EGCG treatment.

## Nutrigenomics Approach

To analyze for the action of EGCG on the expression of genes of intestine and liver, we performed microarray analysis according to the Affymetrix GeneChip Technical Protocol (Thermo Fisher Scientific, Walthman, MA) using a Affymetrix GeneChip Mouse Gene 1.0 ST array. Affymetrix GeneChip Command Console (AGCC) software was used to reduce the array images to the intensity of each probe (CEL files). CEL files were quantified with the quantile Factor Analysis for Robust Microarray Summarization (qFARMS) algorithm using statistical language R and Bioconductor. Differentially expressed genes (DEGs) were identified by applying the Rank Products method. Probe sets presenting a false discovery rate (FDR) <0.05 were regarded as having significantly different expression levels between the two groups. In the liver, HFD intake increases the genes expression related to lipid synthesis, lipolysis, lipid transport, gluconeogenesis, and death of cancer cells. Conversely, the EGCG intake reverses the aforementioned changes. Further, the possibility of ElF2 signaling pathways activation and sirtuin signaling pathways upon EGCG intervention are described (Table 1). Whereas in the colon, the HFD intake suppresses the genes expression related to lipid oxidation, death of cancer cells, as well as sirtuin signaling pathways and the EGCG intake deregulates all of these changes (Table 2).

Sirtuins are highly conserved nicotinamide adenine dinucleotide (NAD^+^)-dependent protein deacetylases and/or ADP-ribosyltransferases that can extend the lifespan of several lower model organisms including yeast, worms and flies. The seven mammalian sirtuins, SIRT1 to SIRT7, have emerged as key metabolic sensors that directly link environmental signals to mammalian metabolic homeostasis and stress response. Recent studies have shed light on the critical roles of sirtuins in mammalian energy metabolism in response to nutrient signals,^(27)^ In addition, NAD⁺ homeostasis is emerging as a key player in the pathogenesis of NAFLD and is tightly linked to the SIRT1/5'-AMP-activated protein kinase (AMPK) pathway.^(28)^ SIRT1 is known to be involved in gluconeogenesis and fatty acid oxidation in the liver. Our data showed that the sirtuin signaling pathway was down-regulated in HFD-fed mice and up-regulated in EGCG-fed mice in the liver as well as intestine. Recently, Bae *et al.* ^(29)^ have also reported the role of SIRT1 and AMPK in the pathogenesis of NAFLD and the effects of EGCG on this pathway. They clearly demonstrated that SIRT1 expression and AMPK phosphorylation were significantly decreased after HFD feeding in the liver, and that GTE supplementation restored SIRT1 expression and the phosphorylation levels of AMPK.^(29)^ Taken together, recent data including ours demonstrate that EGCG exerts anti-steatotic effects through the activation of SIRT1-AMPK pathway.

Recent studies have revealed an intriguing association between the circadian clock and cellular metabolism and have linked SIRT1 function to the regulation of the circadian rhythm. The CLOCK-BMAL1 transcript complex directly controls the expression of NAMPT, which encodes the rate-limiting enzyme in NAD^+^ biosynthesis. NAD^+^ then regulates SIRT1 activity and modulates CLOCK-BMAL1-mediated transcription. SIRT1 also directly regulates the activity of PER, a negative regulator of CLOCK-BMAL1 transcription, through deacetylation.^(30)^ Mi *et al.*^(31)^ reported that HFFD treatment partially exhibited poor circadian oscillations of the core clock gene and the clock-controlled gene in the liver and fat relative to the control group, and that EGCG administration ameliorated the diet-dependent decline in circadian function by controlling the SIRT1-PGC1α loop, implying the existence of an EGCG-entrainable oscillator. This is the first report to demonstrate the enhancement of decline in peripheral type of clock genes by the EGCG ingestion. In addition, Govindarajan *et al.*^(32)^ have demonstrated that unconjugated BAs, known to be generated through the BSH activity of the gut microbiota, are potentially chronobiological regulators of host circadian gene expression, especially in the intestine and liver. These data may indicate the potential role for microbe-generated BAs as chronological regulators of the peripheral circadian clock and suggest that the intervention strategies which alter gut BA profile have the potential to influence the circadian clock. Judging from updated data including ours, EGCG may be a candidate to regulate peripheral circadian clock.

## Conclusion

We summarized recent data demonstrating that microbiota and their metabolites, especially BAs, play a crucial role in the pathogenesis of NAFLD, and that EGCG could exert preventive effect against NAFLD via modulating gut microbiota and host gene expression.

## Figures and Tables

**Table 1:** Down- and up-regulated canonical pathway-related gene expression in the liver of mice fed a high-fat diet (HFD), and effects of EGCG on these changes

Canonical pathway	HFD vs Cont			EGCG vs HFD	
	Activation z-score	–Log (*p* value)		Activation z-score	–Log (*p* value)
Acetone degradation I (to methylglyoxal)	–**2.3**	9.6			
Aryl hydrocarbon receptor signaling	**2.0**	5.1			
Bupropion degradation	–**2.5**	8.2			
EIF2 signaling	–**3.3**	22.8		**2.9**	20.7
Fatty acid β-oxidation I	**3.7**	11.2			
Gluconeogenesis I	**2.2**	2.6		–**2.0**	2.0
Glutaryl-CoA degradation	**2.1**	8.0			
Glycolysis I				–**2.2**	2.9
Isoleucine degradation I				–**2.4**	5.3
Ketogenesis	**2.6**	7.7			
Ketolysis	**2.0**	3.7		–**2.0**	4.0
LPS/IL-1 mediated inhibition of RXR function	–**2.7**	20.6			
Melatonin degradation I	–**3.2**	13.2			
Nicotine degradation II	–**3.1**	15.2			
Nicotine degradation III	–**3.2**	13.0			
Noradrenaline and adrenaline degradation				–**2.1**	5.3
NRF2-mediated oxidative stress response	**2.6**	4.4			
Oleate biosynthesis II (animals)	**2.0**	3.1			
Sirtuin signaling pathway				**2.4**	4.5
Stearate biosynthesis I (animals)	**2.3**	6.9			
Sucrose degradation V (mammalian)				–**2.0**	4.0
Superpathway of cholesterol biosynthesis	**2.4**	18.4			
Superpathway of geranylgeranyldiphosphate biosynthesis I (via mevalonate)	**2.1**	7.1			
Superpathway of melatonin degradation	–**3.2**	12.2			
Triacylglycerol biosynthesis	**2.3**	4.8			
Urea cycle	–**2.0**	4.4			
Valine degradation I				–**2.4**	4.6
γ-Linolenate biosynthesis II (animals)	**2.0**	2.6			

**Table 2:** Down- and up-regulated canonical pathway-related gene expression in the intestine of mice fed a high-fat diet (HFD), and effects of EGCG on these changes

Canonical pathway	HFD vs Cont			EGCG vs HFD	
	Activation z-score	–Log (*p* value)		Activation z-score	–Log (*p* value)
Adenosine nucleotides degradation II				–**2.0**	2.5
Apelin adipocyte signaling pathway	–**2.3**	1.8			
Cholesterol biosynthesis I	–**2.0**	2.5		–**2.2**	3.5
Cholesterol biosynthesis II (via 24,25-dihydrolanosterol)	–**2.0**	2.5		–**2.2**	3.5
Cholesterol biosynthesis III (via desmosterol)	–**2.0**	2.5		–**2.2**	3.5
Cysteine biosynthesis III (mammalia)	–**2.0**	1.7			
EIF2 signaling	**5.1**	30.3			
Glutathione-mediated detoxification	–**2.4**	3.1		–**2.4**	3.1
Glycolysis I				–**2.0**	1.6
NAD salvage pathway II				–**2.0**	1.7
NRF2-mediated oxidative stress response	–**2.4**	3.6			
Oxidative phosphorylation	**2.5**	1.6			
Sirtuin signaling pathway	–**2.6**	4.0		**2.5**	1.6
SPINK1 pancreatic cancer pathway	–**2.8**	7.5		**2.8**	7.6
Superpathway of cholesterol biosynthesis				–**2.5**	6.4
Th1 pathway				–**2.5**	1.5
Urate biosynthesis/inosine 5'-phosphate degradation				–**2.0**	2.5
Valine degradation I				–**2.0**	2.0
